# Short- and long-term outcomes for primary anastomosis versus Hartmann’s procedure in Hinchey III and IV diverticulitis: a multivariate logistic regression analysis of risk factors

**DOI:** 10.1007/s00423-020-02015-6

**Published:** 2020-10-20

**Authors:** Ivan Facile, Raffaele Galli, Pavlo Dinter, Robert Rosenberg, Markus Von Flüe, Daniel Christian Steinemann, Alberto Posabella, Raoul André Droeser

**Affiliations:** 1 Department of Surgery, Cantonal Hospital Baselland, Liestal, Switzerland; 2 Department of Surgery, University Hospital Basel, Spitalstrasse 23, 4031 Basel, Switzerland; 3 Clarunis, Department of Visceral Surgery, University Centre for Gastrointestinal and Liver Disease, St Clara Hospital and University Hospital, Kleinriehenstrasse 30, 4058 Basel, Switzerland

**Keywords:** Complicated diverticulitis, Primary anastomosis, Hartmann’s procedure, Hinchey classification, Stoma rate

## Abstract

**Purpose:**

The management of perforated diverticulitis with generalized peritonitis is still controversial and no preferred standardized therapeutic approach has been determined. We compared surgical outcomes between Hartmann’s procedure (HP) and primary anastomosis (PA) in patients with Hinchey III and IV perforated diverticulitis.

**Methods:**

Multicenter retrospective analysis of 131 consecutive patients with Hinchey III and IV diverticulitis operated either with HP or PA from 2015 to 2018. Postoperative morbidity was compared after adjustment for known risk factors in a multivariate logistic regression.

**Results:**

Sixty-six patients underwent HP, while PA was carried out in 65 patients, 35.8% of those were defunctioned. HP was more performed in older patients (74.6 vs. 61.2 years, *p* < .001), with Hinchey IV diverticulitis (37% vs. 7%, *p* < .001) and in patients with worse prognostic scores (P-POSSUM Physiology Score, *p* < .001, Charlson Comorbidity Index *p* < .001). Major morbidity and mortality were higher in HP compared to PA (30.3% vs. 9.2%, *p* = .002 and 10.6% vs. 0%, *p* = .007, respectively) with lower stoma reversal rate (43.9% vs. 86.9%, *p* < .001). In a multivariate logistic regression, PA was independently associated with lower postoperative morbidity and mortality (OR 0.24, 95% CI 0.06–0.96, *p* = .044).

**Conclusions:**

In comparison to PA, HP is associated with a higher morbidity, higher mortality, and a lower stoma reversal rate. Although a higher prevalence of risk factors in HP patients may explain these outcomes, a significant increase in morbidity and mortality persisted in a multivariate logistic regression analysis that was stratified for the identified risk factors.

## Introduction

The treatment of acute diverticulitis may be conservative or operative, according to the severity of the disease and clinical signs. Conservative management of acute diverticulitis in Hinchey stages I and II with antibiotic therapy and, for larger abscesses, percutaneous drainage, relies on wide and clear evidence [1–3]. Despite the operative treatment being considered the standard therapy for patients with Hinchey stage III or IV diverticulitis, a clear consensus on the best surgical technique is still undetermined [4].

Hartmann’s resection (HP), becoming popular in the 1970s, is considered the more traditional surgical approach, and is still widely practiced [5, 6]. However, Hartmann’s reversal is a challenging procedure associated with high rate of intra- and postoperative morbidity [7]. Reports show that in 30–40% of patients receiving the HP operation, are not considered for stoma closure. This is a result of high surgical risk in older adults and in patients with multiple comorbidities [8, 9].

Due to the disadvantages associated with HP, primary anastomosis (PA) gained popularity from the 1990s, with the further development of surgical techniques [6, 9]. Resection with primary anastomosis has been proven to have the same degree of safety as the Hartmann’s procedure, in terms of postoperative outcome, furthermore the reversal of a diverting loop ileostomy is technically easier with a lower morbidity rate compared to a reversal of Hartmann’s procedure [10, 11].

Current evidence for management of patients with Hinchey III and IV diverticulitis relies mostly on reviews, meta-analysis, and small observational studies, due to the difficulty of randomization of patients with a life-threatening condition. So far only 4 published RCTs compared HP to PA in Hinchey III and IV diverticulitis [12–15]. In all these studies, the results were similar, favoring PA compared to HP considering the higher stoma reversal rate and similar mortality and morbidity rate. However, all these works presented flaws in study design and were underpowered, due to recruitment difficulties.

Despite emerging evidence favoring PA, HP remains a valuable option in hemodynamically unstable or high-risk patients [11]. Furthermore, HP is safe and technically feasible even when performed by surgeons in training with limited colorectal experience [16].

The aim of this multicenter study was to assess the postoperative outcome according to the chosen operative strategy (PA vs. HP) in patients with Hinchey stages III and IV perforated diverticulitis taking into consideration risk factors known to affect morbidity and mortality.

## Methods

### Study cohort

From January 2015 until December 2018, all patients that had surgery for complicated colonic diverticular disease with generalized peritonitis (Hinchey III or IV) at one university and two teaching hospitals in Switzerland were included. To ensure an appropriate duration of at least 12 months for the stoma reversal, the follow-up of included patients was extended until December 2019. Data were derived retrospectively, from each hospital’s Department of Surgery’s database. The following data were retrieved: demographic characteristics, comorbidity such as preoperative renal failure (defined as an estimated glomerular filtration rate < 60 mL/min/1.73 m^2^), immunosuppression (defined as the presence of immunological disease or ongoing chemotherapy or the intake of corticosteroids > 5 mg prednisone per day or equivalent), diabetes, chronic obstructive pulmonary disease (COPD), body mass index (BMI), and number and type of medications. Intraoperative data such as Hinchey classification of perforated diverticulitis (stage III or IV), operating time, blood loss, surgical experience (general surgeon vs. surgeon with subspecialty in abdominal or colorectal surgery) were also acknowledged. Postoperative outcomes including morbidity, mortality within 30 days, length of hospital stay, and intensive care unit (ICU) stay were also recorded. For all patients, the estimated morbidity and mortality according to the P-POSSUM score and the Charlson Comorbidity Index (CCI) were calculated.

Patients were divided into two groups: HP and PA (with or without diverting ileostomy). The type of procedure was determined by the surgeon’s preference, taking into account the patient’s health and considering the intraoperative condition.

### Primary and secondary endpoint

The primary endpoint was mortality within 30 days and major morbidity during in-hospital treatment. Postoperative morbidity was classified according to the Clavien-Dindo system and defined as major if it was greater or equal to 3b [17–19]. Among other complications, we analyzed separately the superficial incisional, deep incisional, and organ space surgical site infections according to the Centers for Disease Control National Nosocomial Infections Surveillance System [20] as well as anastomosis insufficiency or rectal stump leakage. Operating time, blood loss, hospital stay, and ICU stay were secondary endpoints. Restoration of bowel continuity after HP or PA with diverting ileostomy was also reviewed.

### Ethical considerations

This study was approved by the research ethics committee of Nordwestschweiz (EKNZ-2019-01635).

### Statistical analysis

Categorical variables are reported as frequencies (numbers and percentages) and continuous variables as means ± standard deviation. The primary endpoint (major complications; Clavien-Dindo ≥ 3b) was analyzed by uni- and multivariate logistic regression analysis, in the multivariate logistic regression we adjusted for known risk factors such as age, BMI, gender, Hinchey stadium, surgical experience, and comorbidities of the patients (Charlson Comorbidity Index, P-POSSUM Physiology Score, polypharmacy). Referring to existing literature, we defined polypharmacy as the concurrent use of ≥ 5 different prescribed drugs [21]. A *p* value of less than 0.05 was considered statistically significant. All analyses were performed with STATA 13 (StataCorp LP, College Station, TX, USA).

## Results

From January 2015 to December 2018, a total of 131 consecutive patients underwent emergency surgery for perforated diverticulitis with diffuse purulent or fecal peritonitis (Hinchey III or IV). Baseline characteristics, Hinchey stage, and prognostic scores are shown in Table 1.

**Table 1:** Baseline characteristics, Hinchey Stage, surgeon’s experience, and prognostic scores of patients according to the chosen surgical technique (HP vs. PA)

Characteristic	Hartmann’s procedure (*n* = 66)	Primary anastomosis (*n* = 65)	*P* value
Age, year, mean (± SD)	74.6 (± 11.8)	61.2 (± 12.9)	< .001
Gender (M/F), *n* (%)	27 (40.9%)/39 (59.1%)	41 (63.1%)/24 (36.9%)	.011
BMI*, kg/m^2^, mean (± SD)	26.9 (± 5.6)	25.9 (± 4.0)	.634
Hinchey stage IV, *n* (%)	25 (37.8%)	5 (7.5%)	< .001
Preoperative renal failure, *n* (%)	29 (43.9%)	6 (9.2%)	< .001
Immunosuppression, *n* (%)	21 (31.8%)	6 (9.2%)	.005
Diabetes, *n* (%)	10 (15.1%)	2 (3%)	.017
COPD†, *n* (%)	12 (18.1%)	3 (4.6%)	.015
Nr. medications, mean (± SD)	6.1 (± 3.7)	2.9 (± 3.0)	< .001
Anti-aggregation, *n* (%)	14 (21.1%)	10 (15.3%)	.389
Anticoagulation, *n* (%)	22 (33.3%)	8 (12.3%)	.004
Surgeon’s experience, *n* (%)			
General surgeon	36 (54.5%)	27 (41.5%)	.136
Subspecialty in abdominal or colorectal surgery	30 (45.5%)	38 (58.6%)	
P-POSSUM Physiology Score, mean (± SD)	25.6 (± 7.4)	19.2 (± 4.7)	< .001
P-POSSUM Score estimated morbidity, mean % (± SD)	83.4% (± 13.1%)	68.8% (± 13.0%)	< .001
P-POSSUM Score estimated mortality, mean % (± SD)	22.1% (± 20.4%)	8.4% (± 11.3%)	< .001
Charlson Comorbidity Index	5.7 (± 2.8)	2.3 (± 1.9)	< .001

*BMI, body mass index; †*COPD*, chronic obstructive lung disease

Regarding the distribution of the performed HP and PA, there was not a significant difference between our 3 institutions (*p* = 0.568). In the PA group, 29 patients had a laparoscopic procedure and the conversion rate was 51.7%. The main causes for conversion were insufficient control of the sepsis source and inability to identify anatomy because of severe inflammatory changes or extensive adhesions. In 23 cases (35%), an ileostomy was created. In the HP group, 7 laparoscopic approaches were performed, of which 4 needed a conversion to open.

Patients undergoing HP were significantly older (74.6 ± 11.8 vs. 61.2 ± 12.9 years, *p* < 0.001) and Hinchey IV was significantly more frequent compared to the PA group (37.8% vs. 7.5%, *p* < 0.001). Furthermore, the comorbidity rate was higher among HP patients, in particular considering preoperative renal failure (43.9% vs. 9.2%, *p* < 0.001), immunosuppression (31.8% vs. 9.2%, *p* = 0.005), diabetes mellitus (15.1% vs. 3.0%, *p* = 0.017), and chronic obstructive pulmonary disease (18.1% vs. 4.6%, *p* = 0.015). In addition, patients who underwent HP had a higher number of medications (6.1 ± 3.7 vs. 2.9 ± 3.0, *p* < 0.001). The rate of patients with anticoagulant therapy was significantly higher in the HP group compared to the PA group (33.3% vs. 12.3%, *p* = 0.004), while there was no difference regarding anti-aggregation agents (21.1% vs. 15.3%, *p* = 0.389). The calculated prognostic scores showed also a higher comorbidity rate of the HP cohort, as demonstrated by P-POSSUM Physiology Score (25.6 ± 7.4 vs. 19.2 ± 4.7, *p* < 0.001), and the estimated morbidity (83.4% ± 13.1 vs. 68.8% ± 13.0, *p* < 0.001) as well as mortality score (22.1% ± 20.4 vs. 8.4% ± 11.3, *p* < 0.001) according to P-POSSUM. Finally, the Charlson Comorbidity Index was also significantly higher in the HP group compared to the PA group (5.7 ± 2.8 vs. 2.3 ± 1.9, *p* < 0.001). No significant differences were found regarding BMI (26.9 ± 5.6 vs. 25.9 ± 4.0, *p* = 0.634).

All primary and secondary outcomes are summarized in Table 2. The mortality rate within 30 days was significantly higher in the HP group with 7 deaths compared to no event in the PA group (10.6% vs. 0%, *p* = 0.007). The causes of death were acute respiratory failure in 3 patients, abdominal sepsis in 2 patients, cardiac failure in 1 patient, and an underlying advanced non-abdominal cancer in 1 patient. There were no significant differences between the two groups regarding primary wound closure (72.7% vs. 65.6%, *p* = 0.317), superficial incisional (13.6% vs. 6.1%, *p* = 0.152), deep incisional (1.5% vs. 0%, *p* = 0.319), and organ space surgical site infection (4.5% vs. 4.6%, *p* = 0.367).

**Table 2:** Intra- and postoperative outcomes according to the chosen surgical technique (HP vs. PA)

	Hartmann’s procedure (*n* = 66)	Primary anastomosis (*n* = 65)	*P* value
Laparoscopic procedure, *n* (%)	3 (4.5%)	14 (21.5%)	.004
Conversions to open surgery, *n* (%)	4 (57.1%)	15 (51.7%)	.796
Operating time, min, mean (± SD)	154 (± 50)	177 (± 50)	.005
Blood loss, ml, mean (± SD)	200 (± 204)	194 (± 208)	.684
Hospital stay, day, mean (± SD)	18.8 (± 13.9)	12.5 (± 4.8)	.002
ICU stay, day, mean (± SD)	4.7 (± 6.3)	1.7 (± 2.4)	.001
Major complications ≥ 3b, *n* (%)	20 (30.3%)	6 (9.2%)	.002
Primary wound closure, *n* (%)	48 (72.7%)	42 (65.6%)	.317
Superficial incisional SSI, *n* (%)	9 (13.6%)	4 (6.1%)	.152
Deep incisional SSI, *n* (%)	1 (1.5%)	0 (0%)	.319
Organ/space SSI, *n* (%)	3 (4.5%)	3 (4.6%)	.367
Anastomosis insufficiency or rectal stump leak, *n* (%)	2 (3.0%)	3 (4.5%)	.636
Mortality within 30 days, *n* (%)	7 (10.6%)	0 (0%)	.007
Stoma reversal, *n* (%)	29 (43.9%)	20/23 (86.9%)	< .001

Major complications (≥ 3b according to Clavien-Dindo classification) were 30.3% in the HP and 9.2% in the PA group (*p* = 0.002). In the PA group, the defunctioning stoma does not have any significant influence on the major complications (*p* = 0.593). Nine patients in the HP group as well as 6 patients in the PA group were re-operated due to postoperative complications as reported in Table 3. Two of the patients in the HP group developed a rectal stump leak (2/66 patients, 3.0%). They both needed a surgical revision with lavage for control of the sepsis. The incidence of anastomotic leak was 4.6% (3/65 patients). In one patient with primary ileostomy, the leak could be managed conservatively using only antibiotics, while two other patients needed a surgical revision (overstitching of the anastomosis in one patient with initial diversion, and ileostomy construction in one patient without initial diversion). Regarding ostomy-related complications, 2 patients in the HP group were re-operated, due to the development of a colostomy ischemia and 1 patient in the PA group had to be re-operated because of a parastomal hernia causing small bowel obstruction.

**Table 3:** Re-operation for abdominal complications

	Hartmann’s procedure	Primary anastomosis
Rectal stump leak/anastomosis insufficiency, *n*	2	2
Postoperative abdominal bleeding, *n*	2	1
Stoma ischemia, *n*	2	0
Uncontrolled sepsis, *n*	1	1
Small bowel anastomosis insufficiency, *n*	1	0
Fascial dehiscence, *n*	1	0
Port site hernia, *n*	0	1
Parastomale hernia with ileus, *n*	0	1
Total	9	6

Surgeon’s experience was not found to affect the choice of procedure (*p* = 0.136). Operating time was shorter (154 min ± 50 vs. 177 min ± 50, *p* = 0.005) in the HP group and there were no significant differences regarding blood loss (200 ml ± 204 vs. 194 ml ± 208, *p* = 0.684). Patients who underwent PA had a significantly shorter hospital (12.5 ± 4.8 vs. 18.8 ± 13.9 days, *p* = 0.002) and ICU stay (1.7 ± 2.4 vs. 4.7 ± 6.3 days, *p* < 0.001). Stoma reversal was significantly more frequent after PA (86.9% vs. 43.9%, *p* < 0.001).

In only one case, damage control surgery with an initial resection of the perforated colon was performed, followed by secondary reconstruction with PA 48 h later.

Results from a uni- and multivariate logistic regression analysis of risk factors associated with major complications are shown in Table 4. PA and a lower P-POSSUM Physiology Score were significantly associated with a lower incidence of major complications (OR 0.24, 95% CI 0.06–0.96, *p* = 0.044 and OR 1.19, 95% CI 1.07–1.32, *p* = 0.001, respectively), even after taking into consideration known risk factors such as age, BMI, gender, polypharmacy, Hinchey stadium, surgeon’s experience, and CCI.

**Table 4:** Uni- and multivariate logistic regression analysis for major morbidity (complications ≥ 3b according to the Clavien-Dindo classification)

	Univariate			Multivariate		
	OR	95% CI	*P* value	OR	95% CI	*P* value
Primary anastomosis	0.23	0.08–0.63	.004	0.24	0.06–0.96	.044
Age	1.03	0.99–1.07	.055	0.97	0.92–1.02	.341
BMI*	0.94	0.85 – 1.03	.196	0.91	0.81–1.02	.125
Male gender	1.10	0.46–2.60	.825	1.23	0.40–3.75	.710
Charlson Comorbidity Index	1.25	1.08–1.46	.003	0.98	0.73–1.32	.912
P-POSSUM Physiology Score	1.17	1.08–1.25	> .001	1.19	1.07–1.32	.001
Surgeon’s experience	1.34	0.56–3.18	.510	1.70	0.57–5.04	.335
Polypharmacy	1.62	0.68–3.85	.276	0.66	0.18–2.40	.529
Hinchey stadium (IV vs. III)	1.31	0.49–3.51	.586	0.57	0.15–2.16	.414

*BMI, body mass index

## Discussion

Despite the consistent efforts over the past decades, no consensus exists regarding the best surgical therapeutic strategy for perforated diverticulitis in terms of efficacy or safety, and the optimal approach is still undetermined [22]. HP and PA are the most evaluated and accepted options for surgical treatment. With our work, we want to contribute to the current evidence with a retrospective multicenter study comparing patients’ characteristics affecting choice of procedure and postoperative outcomes independent of known surgical risk factors.

To the best of our knowledge, this is one of the largest series of consecutive patients with Hinchey III and IV diverticulitis, where a comparison of PA and HP was carried out. Regarding the choice of procedure, HP was performed more frequently in Hinchey stage IV, in older patients and in patients with significantly higher comorbidities and worse prognostic scores. Similar results were also demonstrated in other retrospective and prospective studies for surgical treatment of complicated diverticulitis, being HP the preferred therapeutic approach in aged and more compromised patients [23–27].

The overall mortality rate was 5.3% and significantly higher in the HP group (10.6% vs. 0%). Our results are in line with those of a meta-analysis that included 3546 patients in 22 observational studies and 3 RCTs, published in 2019 by Halim et al. [28]: in this meta-analysis, overall mortality was lower in the PA group than in the HP group across the observational studies (OR 0.60, 95% CI 0.38–0.95, *p* = 0.03) but not in the meta-analysis of the RCTs (OR 0.44, 95% CI 0.14–1.34, *p* = 0.15). The most recent randomized controlled trial by Lambricht et al., which was not included in the aforementioned meta-analysis, reported similar outcomes after the index procedure and overall morbidity was only different after the stoma reversal procedure. A 12-month stoma-free survival, which was the primary endpoint in this study, was superior in the PA group. This result is not surprising, and it raises the possibility that stoma closure in HP patients was not considered because of fear of operative complications [15]. Another meta-analysis by Cirocchi et al. in 2013 reported lower mortality and morbidity rates following PA, but recommended caution in the interpretation of such results due to limitations in the quality of the included studies and their heterogeneity [29].

As expected and shown in the literature, the stoma reversal rate was significantly higher in the PA group. Several studies showed that patients who underwent PA with ileostomy were more likely to be stoma-free with lower morbidity and mortality related to the stoma reversal procedure in comparison with HP [12, 13, 30].

After HP, rectal stump leak is a severe complication, often requiring an operative revision and may be underestimated; in literature, pelvis sepsis rate after rectal stump complication varies from 3 until 33% [31]. In our series, both rectal stump leakages developed in elderly and frail patients after HP for Hinchey IV diverticulitis. The incidence of anastomotic leak in our study was 4.6% and was similar to that found in the literature [32]. In all these cases, the patients were not immunosuppressed and no intraoperative complications were documented.

In our study, a defunctioning ileostomy was mainly performed based on the surgeon’s experience. Regarding the choice of protecting the anastomosis, we are aware of the fact that our approach may be subject to criticism. Although for rectal anastomosis in low rectal surgery, a defunctioning stoma has been confirmed to reduce intervention rate in the presence of a leak, no definitive consensus has been reported considering the role of proximal diversion in patients with Hinchey III and IV by sigma perforation. In the guidelines of the American Society of Colon and Rectal Surgeons, in the German S2k guidelines for diverticular disease as well as in the WSES Guidelines for the management of acute left-sided colonic diverticulitis it is stated that PA with or without ileostomy is considered the alternative to HP in Hinchey III and IV diverticulitis. On the contrary, in EAES/SAGES consensus, PA with ileostomy represents the only alternative mentioned [4, 33–35]. In our PA group, the use of a proximal diverting stoma did not influence the outcome, but no solid conclusions can be drawn due to the small sample size. Although prospective studies are required to exactly investigate the role of proximal diversion in the PA in Hinchey III and IV patients, we strongly suggest that a defunctioning stoma should be always performed in case of a high-risk anastomosis.

In our cohort, only one patient underwent a damage control surgery with resection and lavage due to preoperative severe sepsis. The patient was discharged after 16 days of hospitalization without any major complications. This anecdotal report does not allow us to advocate for damage control surgery as a recognized strategy in this setting. However, this approach is emerging as an alternative to HP in selected patients with ongoing severe sepsis as stated in the WSES guidelines about management of intra-abdominal sepsis and further efforts are needed to build the necessary evidence [35].

Laparoscopic surgery is routinely practiced for elective colorectal resections in all institutions contributing to this study and even surgeons in training are familiar with minimally invasive procedures. However, laparoscopic treatment of perforated diverticulitis and generalized peritonitis was only performed in 21.5% of PA and 4.5% of HP reflecting the limitations of this technique for an emergency situation in a hostile abdomen [36, 37]. Concerning the high rate of conversion from laparoscopy to open surgery, without any statistical difference between the PA and the HP group (51.7 vs. 57%, *p* = 1, respectively), we would specify that all the patients receiving a laparoscopic approach were patients with stable clinical conditions, while hemodynamically unstable patients always received directly open surgery. To confirm this statement, we performed a statistical analysis of the 15 patients who needed a conversion; the analysis showed that P-POSSUM score and Charlson comorbidity score of those patients were statistically better compared to the critically ill patients requiring a direct open approach (*p* = 0.047 and *p* = 0.026, respectively).

Finally, we would also point out that a laparoscopic approach is feasible only in stable patients and if the surgeon is comfortable with it, taking into consideration in the decision-making process the high probability for conversion. In all hemodynamically unstable patients, the open approach must be the preferred choice.

The primary limitation of this study is its non-randomized study design, which can produce a high selection bias. However, it has been shown that randomization in the life-threatening condition is difficult to apply, mostly due to medical staff’s failure to comply with the randomization protocol, or patients not willing to participate, and that patient selection is a potential concern even in a randomized design, as reported by Binda [12]. In the analysis of his multicenter study, the author complained about the refusal of some surgeons to randomize ill patients, as a result of their skepticism in the applicability of PA in patients with generalized peritonitis [38]. This resulted in poor recruitment and premature termination of the trial, before achieving the planned sample size as highlighted in a meta-analysis by Shaban et al., who stated that the included RCTs were underpowered [32].

In addition, the widespread consensus that, in case of generalized peritonitis, HP should be preferred to PA influenced also our surgeons and should be taken into consideration in the interpretation of our results, representing a selection bias, which can influence the reliability of our conclusions. Despite this, PA was performed in almost 50% of patients, which represents the highest percentage in the literature in comparison to similar observational studies (Trenti et al. 2011: PA in 31% over 87 patients, Gawlick et al. 2012: 13% over 1933 patients, Vennix et al. 2015: 7% over 258 patients) [11, 30, 39].

In order to reduce the bias of the estimate of the treatment effect, we performed a uni- and multivariate logistic regression analysis of risk factors associated with major complications. As shown in our results, PA and a lower P-POSSUM Physiology Score were significantly associated with a lower risk of developing major complications, even after adjusting for these risk factors in the multivariate analysis. The superiority of PA regarding postoperative morbidity in a logistic regression analysis considering Hinchey Stadium, Peritonitis Severity Score, and ASA-Score was described by Trenti et al. [11]. To the best of our knowledge, this is the first study with a significant result in a multivariate analysis taking into consideration surgeons’ experience, comorbidities as well as the clinical condition of the patient on the day of surgery. We suggest that our findings must be interpreted with caution, due to a selection bias, which remains possible based on the retrospective nature of the study design.

To summarize, the results of our study support the recent evidence describing how PA is a valid alternative to HP, not only in patients with a mildly contaminated abdomen but also in the presence of generalized peritonitis, taking into account short-term surgical outcomes and stoma reversal rate. In Europe, there is increasing evidence that favors PA which is slowly accepted by practicing surgeons, even though the preferred therapy is still the non-reconstructive approach. In Northern England, an analysis of 3394 patients with perforated diverticulitis showed a primary anastomosis in only 18% of resections, without statistical changes over the last 14 years [40]. In a recent US-nationwide study, only a few patients underwent an emergency colectomy for diverticulitis with primary anastomosis (7.6%), in the majority of patients (92.4%) Hartmann’s procedure was performed [25]. As suggested by the guidelines of the American Society of Colon and Rectal Surgeons (2020), “the decision to restore bowel continuity should incorporate patient factors, intraoperative factors, and surgeon preference.” [33] However, based on our results and the increasing evidence in the literature, the surgeon’s preference should be no longer a main factor affecting the therapeutic approach, and PA with or without ileostomy should be the preferred option in cardiopulmonary stable patients with perforated diverticulitis.

## Conclusion

There is increasing evidence, including our study, which supports that PA, with or without defunctioning ileostomy, is at least as safe as HP in the treatment of Hinchey III and IV complicated diverticulitis in cardiopulmonary stable patients. Furthermore, patients who undergo PA are more likely to be stoma-free compared to patients who undergo HP. Additional research is warranted to characterize predictive factors for patients with Hinchey III and IV diverticulitis who are at higher risk for anastomotic leakage and might benefit from a diverting loop ileostomy at the time of surgery in case of PA.
